# Knowledge and attitude towards HIV/AIDS in India: A systematic review and meta-analysis of 47 studies from 2010-2020

**DOI:** 10.34172/hpp.2021.19

**Published:** 2021-05-19

**Authors:** Akshaya Srikanth Bhagavathula, Cain C. T. Clark, Rishabh Sharma, Manik Chhabra, Kota Vidyasagar, Vijay Kumar Chattu

**Affiliations:** ^1^Department of Social and Clinical Pharmacy, Faculty of Pharmacy, Charles University, Hradec Kralova, Czech Republic; ^2^Centre for Intelligent Healthcare, Coventry University, Coventry, CV1 5FB, United Kingdom; ^3^Department of Pharmacy Practice, Indo Soviet Friendship College of Pharmacy, Moga, Punjab, India; ^4^Department of Pharmaceutical Sciences, University College of Pharmaceutical Sciences, Hanamkonda, Telangana, India; ^5^Department of Medicine, Temerty Faculty of Medicine, University of Toronto, Toronto, ON M5S 1A8, Canada; ^6^Global Institute of Public Health, Thiruvananthapuram, Kerala, PIN-695024, India

**Keywords:** Human immunodeficiency virus, Acquired immunodeficiency syndrome, Knowledge, Attitudes, India

## Abstract

**Background:** Several studies assessed the level of knowledge and general public behavior on human immunodeficiency virus/acquired immuno-deficiency syndrome (HIV/AIDS) in India. However, comprehensive scrutiny of literature is essential for any decision-making process. Our objective was to perform a systematic review and meta-analysis to examine the level of knowledge and attitude towards HIV/AIDS in India.

**Methods:** A systematic search using Medical Subject Headings (MeSH) and free terms was conducted in PubMed/Medline, Scopus, Embase, and Google Scholar databases to investigate the level of knowledge and attitude of HIV/AIDS in India population. Cross-sectional studies published in English from January 2010 to November 2020 were included. The identified articles were screened in multiple levels of title, abstract and full-text and final studies that met the inclusion criteria were retrieved and included in the study. The methodological quality was assessed using the Joanna Briggs Institute’s checklist for cross-sectional studies. Estimates with corresponding 95% confidence intervals (CIs) for each domain were pooled to examine the level of knowledge and attitude towards HIV/AIDS in India.

**Results:** A total of 47 studies (n= 307 501) were identified, and 43 studies were included in the meta-analysis. The overall level of knowledge about HIV/AIDS was 75% (95% CI: 69-80%; I2 = 99.8%), and a higher level of knowledge was observed among female sex workers (FSWs) 89% (95% CI: 77-100%, I2 = 99.5%) than students (77%, 95% CI: 67-87%, I2 = 99.6%) and the general population (70%, 95% CI: 62-79%, I2 = 99.2%), respectively. However, HIV/AIDS attitude was suboptimal (60%, 95% CI: 51-69%, I2 = 99.2%). Students (58%, 95% CI: 38-77%, I2 = 99.7%), people living with HIV/AIDS (57%, 95% CI: 44-71%, I2 = 92.7%), the general population (71%, 95% CI: 62-80%, I2 = 94.5%), and healthcare workers (HCWs) (74%, 95% CI: 63-84%, I2 = 0.0%) had a positive attitude towards HIV/AIDS. The methodological quality of included studies was "moderate" according to Joanna Briggs Institute’s checklist. Funnel plots are asymmetry and the Egger’s regression test and Begg’s rank test identified risk of publication bias.

**Conclusion:** The level of knowledge was 75%, and 40% had a negative attitude. This information would help formulate appropriate policies by various departments, ministries and educational institutions to incorporate in their training, capacity building and advocacy programs. Improving the knowledge and changing the attitudes among the Indian population remains crucial for the success of India’s HIV/AIDS response.

## Introduction

Over three decades, HIV/AIDS infected around 37.9 million people globally and is a major public health problem.^1^ HIV/AIDS is the second most infectious disease globally, and India has the third largest HIV epidemic in the world.^2^ Since 1992, the National AIDS Control Organization (NACO), under the Ministry of Health and Family Welfare, took several phases of National AIDS Control Programmes (NACP) to improve public knowledge, awareness, and attitudes, as a part of the public health prevention and treatment programs.^3^ Over the preceding two decades, four phases of NACP have been implemented, and most recent reports suggest that the annual number of new HIV infections has decreased by 66%, and death rate by 54%, in India.^3^

Since the inception of HIV/AIDS, the only way to fight against this infectious disease is to increase awareness, knowledge, and modify general public’s behavior. Therefore, a lack of awareness, poor knowledge about various aspects of the disease, and negative perceptions can affect preventive initiatives to control HIV/AIDS. In India, the HIV/AIDS epidemic is highly heterogeneous, and dynamics in population, cultures, level of education, religion issues, and societies are frequently reported barriers that can affect an individual’s knowledge and attitude towards HIV/AIDS.^4-7^ Several studies have been carried out to investigate the level of knowledge and attitude towards HIV/AIDS in India.^8-54^ Indeed, since 2010, much evidence on this topic has been published. However, comprehensive scrutiny to understand the level of knowledge and attitude towards HIV/AIDS among the Indian population has not been conducted. Thus, this study sought to systematically review and quantitatively estimate the current level of knowledge and attitude towards HIV/AIDS in India.

## Materials and Methods

This systematic review and meta-analysis were conducted in accordance with the Preferred Reporting Items for Systematic Review and Meta-Analysis (PRISMA) guidelines.^55^ Cross-sectional observational studies, conducted in India, and published between January 1, 2010, to November 30, 2020, were considered and our search was initiated on April 10, 2020 until December 5, 2020.

### Literature search

A literature search was conducted using a combination of the text and Medical Subject Headings (MeSH) keywords in four databases: PubMed/Medline, Scopus, Embase, and Google scholar, to identify peer-reviewed publications. Several keywords were used, such as; knowledge* OR attitude*, AND cross-sectional studies*, AND questionnaire*, AND surveys*, AND observational* AND sexually-transmitted diseases*, AND human immunodeficiency virus* OR HIV*, AND acquired immunodeficiency syndrome* OR AIDS*, AND physicians* OR doctors* OR primary care* OR dentists* OR dental* OR nurses* OR nursing* OR community health workers* OR public health nursing* OR health professionals* OR public health* OR pharmacy* OR medical students* OR nursing students* OR dental students* OR school students* OR population* OR community*, AND India*. A detailed list of keywords used to identify the literature is presented in Table S1 (Supplementary file 1). The field was limited to “title/abstract,” and the type of publication was limited to “original articles” or “full-length research articles”. We excluded interventional studies, letters, case reports, study protocols, reviews, opinions, grey literature, and non-peer-reviewed publications. The reference lists of articles were also examined to identify other potentially relevant articles. Surveys using open-ended questions focusing on knowledge and attitude about HIV/AIDS were considered. No published or in-progress systematic review on this topic was identified in the Cochrane Library and PROSPERO before this review. The protocol for this systematic review and meta-analysis has been registered in PROSPERO 2019 (CRD42019140447).^56^

### Selection of studies

Two researchers (AB and CC) independently screened the titles and abstracts to identify potentially eligible studies, and further assessment was performed by three authors (RS, MC and KV). Only full-text papers available in the English were included. Small changes in the wording were also disregarded to understand their exact functional meaning. The authors excluded duplicates and studies conducted outside India.

### Data extraction

The extracted data included the name of authors, year of publication, study design, study location, sampling, methods of administration of the questionnaire, and main results. All these details were captured and recorded in an Excel sheet. The information reported in or calculated from the included studies was used for analysis. Corresponding authors were not contacted for unpublished or additional information. Disagreements related to the inclusion of a study were resolved through consensus amongst the authors.

### Quality assessment

Methodological quality and risk of bias of each study were assessed using the Joanna Briggs Institute’s checklist for critical appraisal,^57^ which comprises a nine-item checklist to evaluate whether the sample is representative of the target population. Questions include the following: were the study participants recruited appropriately?; was sample size adequate?; were the study subjects and settings described precisely?; was the data analysis used to identify the sample?; were objectives and standard criteria used to measure the condition?; and were important confounders identified or considered? The studies’ methodological quality was also assessed using the Strengthening the Reporting of Observational Studies in Epidemiology (STROBE) scale.^58^

### Statistical analysis

Meta-analysis was performed using STATA version 16 software (STATA Corporation, College Station, Texas 77, 845 USA). The heterogeneity of the studies was evaluated using Cochrane’s Q-test and I^2^ statistics. We used DerSimonian and Laird’s random-effect model was used to calculate the overall and pooled effect size. Forest plots were used to demonstrate the selected studies in terms of estimates and presented as proportion (%) with corresponding 95% confidence intervals (CIs). Meta-regression was performed to identify the cause of heterogeneity in the year of publication. The differences in the knowledge and attitude across various study groups were assessed using subgroup analysis. The sensitivity analysis was conducted to evaluation effect of each study on the combined result and publication bias was assessment with the funnel plot, “trim and fill” method, Begg’s and Egger’s test. Furthermore, studies were stratified based on high quality (over 75% of the STROBE checklist) and low quality (under 75% of the STROBE checklist). A two-tailed *P*value of less than 0.05 was considered statistically significant.

## Results

**Figure 1:**
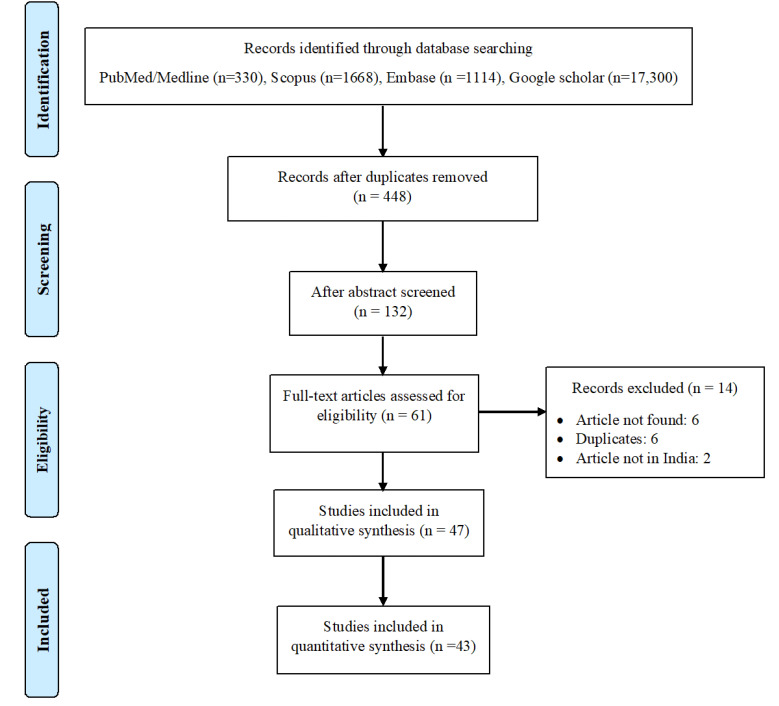
Flow of information through different phases of the systematic review.

A total of 20 412 studies were obtained through database searching; after excluding irrelevant titles and duplicate records, a total of 132 abstracts were considered for screening. Of these, sixty-one studies were considered for the full-text review, and 14 were excluded for various reasons (Table S2, Supplementary file 1). Lastly, 47 studies^8-54^ were considered for the systematic review, and 43 were included in the meta-analysis (Figure 1).^8-15,17-21,23-29,31-52,54^

### Study characteristics

**Figure 2:**
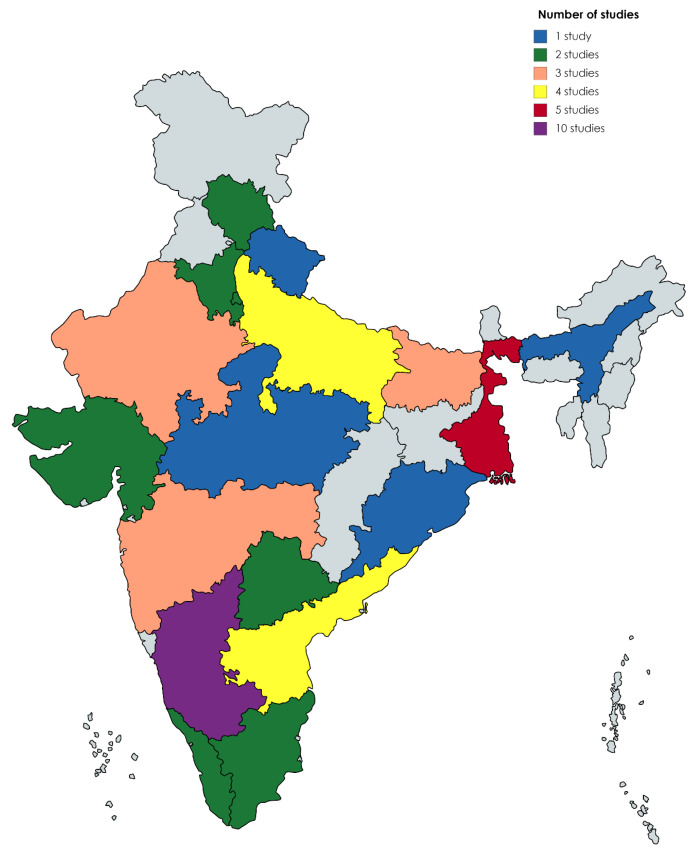
Number of studies reporting knowledge and attitude towards HIV/AIDS in India by state.

The studies included in the systematic review were cross-sectional observational studies using face-to-face or self-administered questionnaires, published between January 1, 2010 to November 30, 2020. A total of forty-seven studies,^8-54^ comprising 307 501 participants, were included, and the number of studies reporting knowledge and attitude about HIV/AIDS in India, by state, is shown in Figure 2. These studies come from most of the Indian states with Karnataka state having ten studies included in the current review.

The sample sizes ranged from 36^23^ to 132 678.^52^ The primary target population across studies were students (n=19),^10,14,15,17,21,23,26,27,29,31,34,35,37,38,40,42,46,47,49^ general population (n=9),^9,13,18-20,43,48,51,52^ healthcare workers [HCWs] (n=5),^8,24,28,41,50^ people living with HIV (PLWHIV) (n=4),^25,32,33,36^ and female sex workers (FSWs) (n=3).^11,12,45^ More details are reported in Table 1.

**Table 1:** Core characteristics of the studies included in Systematic review and Meta-analysis

**Author**	**Year**	**Study design**	**Study location**	**Quality assessment**	**Sample size**	**Focusing group**	**Questionnaire administration**	**Outcome**	**Quality** ^a^	**References**
Ghosh et al	2020	Cross-sectional, questionnaire-based survey	Kolkata	<75%	250	Nurses	Face-to-face	Positive knowledge	6	^8^
Joshi et al	2020	Cross-sectional, questionnaire-based survey	Jodhpur	<75%	1200	Slums	Face-to-face	Positive knowledge	6	^9^
Vittal and Murthy	2020	Cross-sectional, questionnaire-based survey	Andhra Pradesh	<75%	234	Medical and nursing students	Self-administered	Poor knowledge and positive attitude	3	^10^
Kumar	2020	Cross-sectional, questionnaire-based survey	Uttar Pradesh	<75%	195	Female sex workers	Face-to-face	Positive knowledge	5	^11^
Sinha et al	2020	Cross-sectional, questionnaire-based survey	Alipurduar	<75%	90	Female sex workers	Face-to-face	Positive knowledge, negative attitude and positive practice	6	^12^
De Souza et al	2019	Cross-sectional, questionnaire-based survey	Mangalore	<75%	1535	Students, teachers and parents	Self-administered	Modest knowledge	6	^13^
Saheer et al	2019	Cross-sectional, questionnaire-based survey	Kerala	<75%	341	Dental students	Face-to-face	Positive knowledge, negative attitude	5	^14^
Biswas and Bandyopadhyay	2019	Cross-sectional, questionnaire-based survey	West Bengal	>75%	296	School students	Self-administered	Positive knowledge	4	^15^
Sarkar et al	2019	Cross-sectional, questionnaire-based survey	Kolkata	<75%	220	PLWHIV	Face-to-face	Negative perception	5	^16^
Limaye et al	2019	Cross-sectional, questionnaire-based survey	Mumbai	>75%	199	College students	Face-to-face	Positive knowledge and attitude	5	^17^
Meharda et al	2019	Cross-sectional, questionnaire-based survey	Ajmer	<75%	288	Slums	Face-to-face	Positive knowledge and poor practice	5	^18^
Singh et al	2019	Cross-sectional, questionnaire-based survey	Patna	<75%	120	General population	Self-administered	Positive knowledge and attitude, poor practice	5	^19^
Khandekar and Walvekar	2018	Cross-sectional, questionnaire-based survey	Kangrali	<75%	400	Married men	Face-to-face	Positive knowledge and attitude	6	^20^
Chowdary et al	2018	Cross-sectional, questionnaire-based survey	Guntur	<75%	400	Engineering students	Self-administered	Positive knowledge and attitude	6	^21^
Doda et al	2018	Cross-sectional, questionnaire-based survey	Uttarakhand	>75%	385	Consultants, residents, medical students, laboratory technicians, and nurses	Face-to-face	Positive knowledge, receptive attitude and satisfactory practice	7	^22^
Roy et al	2018	Cross-sectional, questionnaire-based survey	Eastern India	<75%	36	Medical students	Face-to-face	Positive knowledge	5	^23^
Dhanya et al	2017	Cross-sectional, questionnaire-based survey	Trichur district of Kerala	<75%	206	Dentists	Self-administered	Positive knowledge, attitude and practice	6	^24^
Banagi Yathiraj et al	2017	Cross-sectional, questionnaire-based survey	Manglore, Karnataka	<75%	409	PLWHIV	Face-to-face	Poor knowledge	7	^25^
Subbarao and Akhilesh	2017	Cross-sectional, questionnaire-based survey	Bengaluru and others	<75%	350	Engineering students	Face-to-face	Positive knowledge and negative attitude	5	^26^
Rahman and Santhosh Kumar	2017	Cross-sectional, questionnaire-based survey	Chennai	<75%	100	Undergraduate students	Self-administered	Positive knowledge and negative attitude	6	^27^
Ngaihte et al	2017	Cross-sectional, questionnaire-based survey	Delhi, Gandhinagar, Bhubaneswar, and Hyderabad	<75%	503	Dentists	Face-to-face	Positive knowledge and modest attitude	6	^28^
Kalyanshetti and Nikam	2016	Cross-sectional, questionnaire-based survey	Belgavi	<75%	102	Nursing students	Face-to-face	Positive knowledge	5	^29^
Baruah et al	2016	Cross-sectional, questionnaire-based survey	Jorhat	<75%	261	Adolescents	Face-to-face	Unclear	3	^30^
Chaudhary et al	2016	Cross-sectional, questionnaire-based survey	Jaipur	>75%	613	School students	Face-to-face	Positive knowledge	6	^31^
Gupta et al	2016	Cross-sectional, questionnaire-based survey	Himachal Pradesh	<75%	150	PLWHIV	Face-to-face	Positive knowledge, positive attitude and negative perception	5	^32^
Bhagavathula et al	2015	Cross-sectional, questionnaire-based survey	Warangal, Telangana	>75%	542	Family of PLWHIV	Face-to-face	Positive knowledge, modest attitude and positive perception	8	^33^
Kumar et al	2015	Cross-sectional, questionnaire-based survey	Raichur	<75%	425	Medical and dental students	Self-administered	Positive knowledge and attitude	6	^34^
Gupta et al	2015	Cross-sectional, questionnaire-based survey	Gorakhpur, Uttar Pradesh	<75%	250	Technical institute students	Face-to-face	Positive knowledge	6	^35^
Mittal et al	2015	Cross-sectional, questionnaire-based survey	Karnataka, Davangere	<75%	100	PLWHIV	Face-to-face	Poor knowledge and attitude	6	^36^
Dubey et al	2014	Cross-sectional, questionnaire-based survey	North India	<75%	630	College students	Face-to-face	Positive knowledge and attitude	7	^37^
Grover et al	2014	Cross-sectional, questionnaire-based survey	NCR	<75%	600	Dental students	Face-to-face	Positive knowledge and attitude	5	^38^
Jogdand and Yerpude	2014	Cross-sectional, questionnaire-based survey	Guntur	<75%	138	Medical students	Face-to-face	Positive knowledge	6	^39^
Oberoi et al	2014	Cross-sectional, questionnaire-based survey	NCR	<75%	610	Dental students	Face-to-face	Poor knowledge and positive attitude	5	^40^
Prabhu et al	2014	Cross-sectional, questionnaire-based survey	Tamilnadu	<75%	102	Dentists	Self-administered	Positive knowledge	5	^41^
Sakalle et al	2014	Cross-sectional, questionnaire-based survey	Indore district	<75%	200	School students	Face-to-face	Positive knowledge	6	^42^
Tondare et al	2013	Cross-sectional, questionnaire-based survey	Mumbai	<75%	256	Adolescents	Face-to-face	Positive knowledge and modest attitude	6	^43^
Jindal	2013	Cross-sectional, questionnaire-based survey	Moodbidri	<75%	300	College students	Face-to-face	Positive knowledge	4	^44^
Hemalatha et al	2013	Cross-sectional, questionnaire-based survey	Andhra Pradesh	<75%	5580	Female sex workers	Face-to-face	Positive knowledge	4	^45^
Gupta et al	2013	Cross-sectional, questionnaire-based survey	Lucknow	<75%	215	School students	Face-to-face	Positive knowledge	5	^46^
Aggarwal and Panat	2013	Cross-sectional, questionnaire-based survey	Bareilly	>75%	460	Dental students	Self-administered	Positive knowledge and attitude	6	^47^
Cooperman et al	2013	Cross-sectional, questionnaire-based survey	Mumbai	<75%	300	Women	Face-to-face	Positive knowledge	6	^48^
Fotedar et al	2013	Cross-sectional, questionnaire-based survey	Shimla	>75%	191	Dental students	Self-administered	Positive knowledge and negative attitude	6	^49^
Achappa et al	2012	Cross-sectional, questionnaire-based survey	Manglore	>75%	200	Nurses	Face-to-face	Positive knowledge, attitude and perception	6	^50^
Yadav et al	2011	Cross-sectional, questionnaire-based survey	Saurashtra	>75%	1237	Young population	Face-to-face	Positive knowledge	5	^51^
Hazarika	2010	Cross-sectional, questionnaire-based survey	Rural and urban	<75%	132678	General population	Face-to-face	Positive knowledge and attitude	4	^52^
Jayanna et al	2010	Cross-sectional, questionnaire-based survey	Karnataka	<75%	393	Female sex workers	Face-to-face	Unclear	4	^53^
Taraphdar et al	2010	Cross-sectional, questionnaire-based survey	Kolkata	>75%	90	PLWHIV	Face-to-face	Positive knowledge and attitude	5	^54^

^a^Joanna Briggs Institute’s criteria

### Knowledge about HIV/AIDS

**Figure 3:**
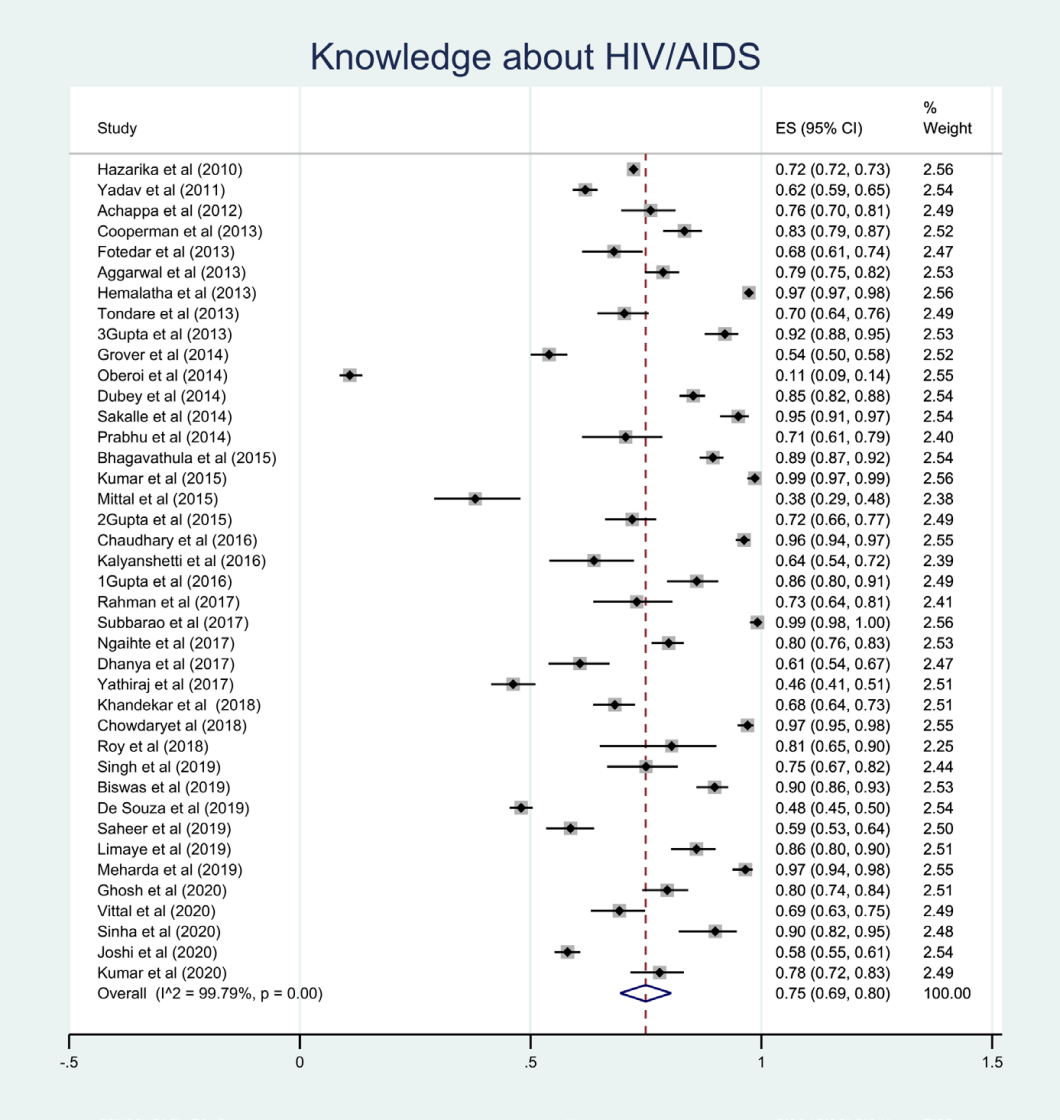
Knowledge about HIV/AIDS.

Forty studies reported on knowledge about HIV/AIDS,^8-15,17-21,23-29,31-38,40-43,45-52^ where the overall level of knowledge was 75% (95% CI: 69-80%, *P* < 0.001) (Figure 3).

The subgroup analysis showed the level of knowledge about HIV/AIDS was high among FSWs (89%),^11,12,45^ while the level of knowledge among PLWHIV was 65%.^25,32,33,36^ Additional information is presented in Table 2.

### Attitude towards HIV/AIDS

**Figure 4:**
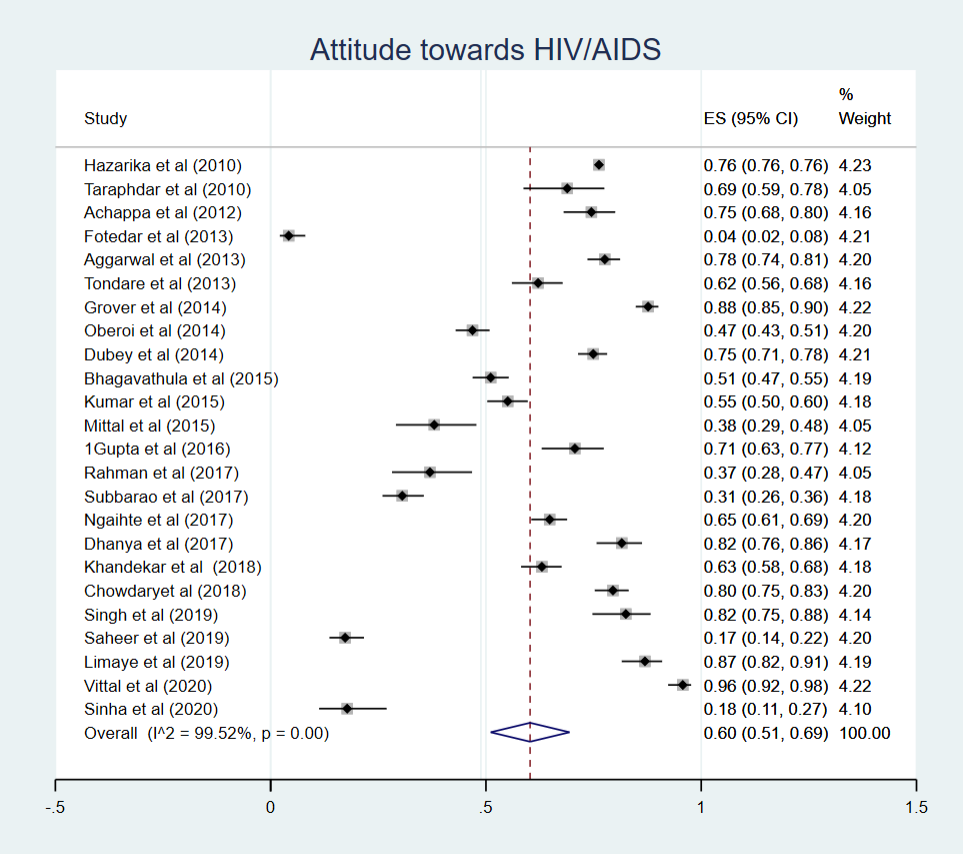
Attitude towards HIV/AIDS.

Twenty-four studies reported the attitude towards HIV/AIDS,^10,12,14,17,19-21,24,26-28,32-34,36-38,40,43,47,49,50,52,54^ where an overall percentage of 60% (95% CI: 51-69%, *P*< 0.001) of subjects had a positive attitude about HIV/AIDS (Figure 4).

Subgroup analysis showed that HCWs,^24,28,50^ as well as general population,^19,20,43,52^ had a positive attitude towards HIV/AIDS, with 74% (95% CI: 63-84%) and 71% (95% CI: 62-80%), respectively. However, only one study investigated the level of attitude about HIV/AIDS in FSWs^12^ and reported only 18% (95% CI: 11-27%). More information is presented in Table 2.

### Meta-regression

Meta-regression based on the year of publication was considered to understand the influence of each study on the overall effect size. Meta-regression analysis suggested no influence of year of publication on the knowledge (Coef= - 0.0052, *P*=0.773) and attitude towards HIV/AIDS (Coef= 0.0036, *P* =0.737) (Figure S1, Supplementary file 1).

### Sensitivity analysis

To address the issue of heterogeneity, studies were classified into high (>75%) and low quality (<75%), according to the STROBE checklist for methodological quality. High-quality studies reported higher knowledge about HIV/AIDS than low-quality studies (81% vs 73%). However, no significant difference in the attitude levels was seen between low- and high-quality studies (Table 2). Figure S2 (Supplementary file 1) presented sensitivity analysis for included studies and showed significant differences beyond the limits of 95% CI of calculated combined results.

**Table 2:** Subgroup analysis of Knowledge and attitude towards HIV/AIDS

**Subgroups**	**Knowledge**			**Attitude**		
	**Studies**	**Sample size**	**Estimates (95% CI)**	**Studies**	**Sample size**	**Estimates (95% CI)**
PLWHIV	5	1291	65% (40% - 90%)	4	882	57% (44% - 71%)
Healthcare workers	5	1261	74% (67% - 80%)	3	909	74% (63% - 84%)
Students	19	5366	77% (67% – 87%)	13	4540	58% (38% - 77%)
General public*	10	138 014	70% (62% - 79%)	4	133 454	71% (62% - 80%)
Female sex workers	3	5865	89% (77% - 100%)	1	90	18% (11% - 27%)
Low quality^a^	34	149 293	73% (67% - 80%)	18	104 593	60% (29% - 92%)
High quality^b^	9	3828	81% (71% - 91%)	6	1682	60% (52% - 69%)

^a^<75% response rate and ^b^≥75% response rate. *General population, community residents, school students, prisoners, and pregnant women.

### Study quality assessment

**Figure 5:**
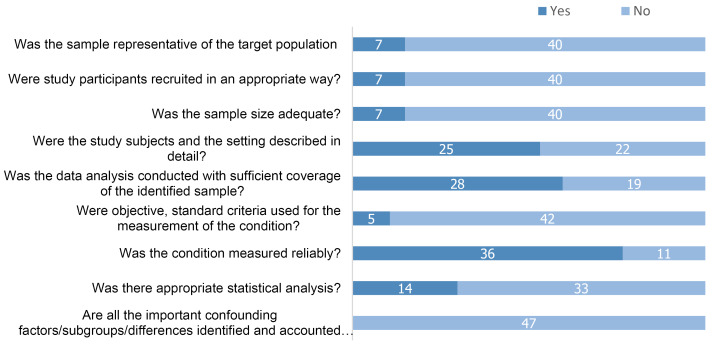
Quality assessment of included studies using the Joanna Briggs Institute’s criteria.

Study quality was evaluated using the Joanna Briggs Institute’s criteria (Figure 5), where a set of nine criteria were used to evaluate the quality of the studies. Seven studies showed that the sample represented the target population,^20,24,25,27,33,51,52^ the participants have been recruited appropriately,^8,11,12,16,18,33,43^ and calculated the sample size.^11,18,20,22,25,33,43^ Twenty-five studies described their study settings,^9,11,12,19,21-25,27,29,31,33-37,39,42,47-50,53,54^ Thirty-two studies conducted the data analysis sufficiently^8,9,13-22,24-28,31-37,39,41-43,48-51^ and five studies used standard criteria to assess HIV/AIDS.^19,22,28,35,48^ The majority of the included studies measured precisely, ^8,12,13,20-29,31-52,54^ 14 studies used appropriate statistical analysis,^9,13,14,17,20,22,25,28,33,37,38,40,46,47^ but none identified major confounders and subgroups.^8-54^

### Publication bias

Publication bias was highlighted in included studies and was confirmed by asymmetric funnel plots. Furthermore, the Begg’s rank test identified a considerable proportion of bias in the knowledge statements (*P* < 0.05) and the Egger’s regression test showed a statistically significant publication bias in the attitude statements related to HIV/AIDS (*P* < 0.05) (Table 3). To reduce this publication bias Trim and fill analysis was conducted and the result was depicted on Figure S3 (Supplementary file 1).

**Table 3:** Risk of bias

	**Egger test**		**Begg’s test**	
	***t-value***	***P value***	***z-value***	***P value***
Knowledge	0.08	0.938	2.34	**0.019**
Attitude	-2.27	**0.033**	1.22	0.224

## Discussion

In the present study, we assimilated studies that assessed knowledge and attitude of HIV/AIDS in India, published from January 2010 to November 2020. To our knowledge, this is the first comprehensive review of this topic. However, some prior reviews have investigated the level of adherence to antiretroviral therapy^59^ and HIV/AIDS-related stigma and discrimination in India.^60^ We identified a total of 47 studies that evaluated the knowledge and attitude of HIV/AIDS in 307 501 participants; accordingly, we were able to perform a series of robust meta-analyses, therein providing a hitherto unreported insight into knowledge and attitude about HIV/AIDS in the Indian population.

Our results are interesting, indeed, as three-quarters (75%) of the subjects had adequate knowledge about HIV/AIDS, but only 60% exhibited a positive attitude. Our findings are consistent with another meta-analysis conducted on an Arabian population where the level of knowledge was 74.4%, and attitude was 53% towards HIV/AIDS, respectively.^61^ Such findings are somewhat lackluster, given the NACP has undertaken several initiatives to increase awareness among the general population by implementing a large number of innovative awareness programs for HIV prevention. For instance, in 2018, NACO initiated multimedia campaigns across television channels, radio broadcastings, online programs, and at cinemas to increase HIV awareness among the general population. A special emphasis was given to HIV testing among the young population.^62^ In 2017, NACO conducted a national survey on the wider Indian population and identified that only one-third of men and one-fifth of women aged between 15-49 had sufficient knowledge of HIV/AIDS.^63^ These findings point to a systematic lack of comprehensive knowledge, prevalent in India, and such deficits in knowledge levels may contribute to false perceptions towards HIV/AIDS. Hence, it is clear that there is much room for improvement in facilitating increases in the basic knowledge about HIV/AIDS among the Indian population through intensive, scientifically guided, educational interventions.

Further, it was observed that the lack of sufficient knowledge reflected negatively on attitudes, and some studies reported more than half of the subjects had a negative attitude towards HIV/AIDS.^12,14,26,27,36,40,49^ The underlying differences in their attitudes are plausibly due to lack of adequate knowledge, negative perception, variations in the sociocultural taboos, and other characteristics that might underlie this negative attitude. For example, a 2016 survey, by the United Nations AIDS study, found that a third of Indian adults had a discriminatory attitude towards PLWHIV, and suggested that activities related to reducing stigma and discrimination are similar to the levels recorded a decade earlier in 2006.^64^ In our subgroup analysis, around 43% of the PLWHIV, 42% of the students, 29% of the general population, and a quarter of HCWs, demonstrated a negative attitude towards HIV/AIDS. Although it is difficult to identify the underlying rationale for these negative attitudes, several studies in India have shown that one-third to half of the respondents, including HCWs. They blame PLWHIV for their infection, endorse denial of their right to marry, and support their isolation from the community.^60,65^ While India made considerable progress in reducing new infections and HIV-related mortality, further efforts are required to change, not only the attitude, but also the pervasive public behaviors, inequalities, societal taboos, stigma, and discrimination towards HIV/AIDS. The wide variations in the knowledge and differences in attitudes reflect the lack of adequate understanding and misconceptions about HIV/AIDS across subgroups. Health administrators and policymakers’ role in providing sufficient training and interventions to level up the awareness and changing the attitude may change the stigma and other inequalities among the HIV/AIDS population.

Although the present study presents a novel addition to the literature, some limitations should be addressed. Firstly, through a comprehensive search strategy, we included 43 cross-sectional observational studies in the meta-analysis and showed high heterogeneity and variations in the responses. This resulted in a significant publication bias, as shown in the asymmetric funnel plots, Begg’s rank test, and Egger’s regression test, respectively. Considering this, only a limited number of studies reported the sample size,^11,18,20,22,25,33,43^ and following the STROBE checklist, we have identified that most of the studies included had low methodological quality. Expecting a high heterogeneity, we used a random-effect model and performed a subgroup analysis to investigate the source of heterogeneity. Secondly, although several comprehensive, validated questionnaires to measure the knowledge and attitude towards HIV/AIDS are freely available,^66-71^ most of the studies did not use validated questionnaires. Thus, because of the non-uniformity of study instruments across the studies, we provided only general observations of knowledge and attitudes of HIV/AIDS. Thirdly, all the studies used self-administered questionnaires, and responses are self-reported; therefore, it is conceivable that responses may overestimate or underestimate the true responses and recall bias. Finally, as the sociodemographic, sociocultural, and geographic variations influence the level of awareness and attitudes, it should be considered in future research.

## Conclusion

The overall knowledge about HIV/AIDS in India was found to be reasonable (75%), with about two-thirds (60%) of those indicating a positive attitude. However, students predominantly had a negative attitude towards HIV/AIDS. This evidence-based information would help formulate appropriate policies by the concerned departments, ministries and educational institutions in India. The government should keep designing effective training, capacity building, and strong advocacy programs to improve the general population’s knowledge levels thereby reducing the false perceptions, stigma, and discrimination towards PLWHIV. Finally, improving the knowledge and changing the attitudes among the Indian population remains crucial for the success of India’s HIV/AIDS response. The study findings will add value to the existing scientific knowledge base not only for India but also at global level in this domain.

## Acknowledgements

The authors thank PROSPERO, National Institute of Health Research for reviewing and approving our study protocol.

## Funding

Nil.

## Competing interests

Vijay Kumar Chattu is an Advisory Board Member for Health Promotion Perspectives. Other authors declare no competing interests.

## Ethical approval

Not applicable.

## Authors’ contributions

AB, CC, RS, MC, KV and VC conceptualized and designed the study. AB and CC independently screened the titles and abstracts to identify potentially eligible studies, and further assessment was performed by three authors RS, MC and KV. AB conducted the statistical analysis and others assisted with data extraction and curation of the database. All authors contributed to the interpretation of data and provided critical inputs in the draft manuscript. VC edited the final draft and all authors have read and approved the final manuscript.

## Supplementary Materials


Online Supplementary file 1 contains Tables S1-S2 and Figures S1-S3.
