# Supplementary material for: Knowledge and attitude towards HIV/AIDS in India: A systematic review and meta-analysis of 47 studies from 2010-2020
Source: Health Promot Perspect. 2021 May 19;11(2):148–60. doi: 10.34172/hpp.2021.19 (PMC8233681; doi:10.34172/hpp.2021.19)

Supplementary file 1

Table S1. Search strategy

PubMed/Medline	Scopus	Embase	Google scholar
(((((("knowledge"[MeSH Terms] OR "knowledge"[All Fields]) OR "knowledge s"[All Fields]) OR "knowledgeability"[All Fields]) OR "knowledgeable"[All Fields]) OR "knowledgeably"[All Fields]) OR "knowledges"[All Fields]) AND ((("attitude"[MeSH Terms] OR "attitude"[All Fields]) OR "attitudes"[All Fields]) OR "attitude s"[All Fields]) AND (((((((((("practicability"[All Fields] OR "practicable"[All Fields]) OR "practical"[All Fields]) OR "practicalities"[All Fields]) OR "practicality"[All Fields]) OR "practically"[All Fields]) OR "practicals"[All Fields]) OR "practice"[All Fields]) OR "practice s"[All Fields]) OR "practiced"[All Fields]) OR "practices"[All Fields]) OR "practicing"[All Fields]) AND (("curr opin hiv aids"[Journal] OR ("hiv"[All Fields] AND "aids"[All Fields])) OR "hiv aids"[All Fields]) AND ((("india"[MeSH Terms] OR "india"[All Fields]) OR "india s"[All Fields]) OR "indias"[All Fields])	(ALL(Knowledge attitude practice HIV AIDS India) AND (LIMIT-TO (PUBYEAR,2020) OR LIMIT-TO (PUBYEAR,2019) OR LIMIT-TO (PUBYEAR,2018) OR LIMIT-TO (PUBYEAR,2017) OR LIMIT-TO (PUBYEAR,2016) OR LIMIT-TO (PUBYEAR,2015) OR LIMIT-TO (PUBYEAR,2014) OR LIMIT-TO (PUBYEAR,2013) OR LIMIT-TO (PUBYEAR,2012) OR LIMIT-TO (PUBYEAR,2011) OR LIMIT-TO (PUBYEAR,2010)) AND (LIMIT-TO (EXACTKEYWORD,"Human") OR LIMIT-TO (EXACTKEYWORD,"Article")) AND (LIMIT-TO (LANGUAGE,"English")))	(('knowledge attitude practice hiv aids india' OR (('knowledge'/exp OR knowledge) AND ('attitude'/exp OR attitude) AND ('practice'/exp OR practice) AND ('hiv'/exp OR hiv) AND ('aids'/exp OR aids) AND ('india'/exp OR india))) AND india OR 'cross-sectional study') AND english:la AND knowledge AND attitude AND practice AND india:ca AND [2010-2020]/py	"hiv" OR "human immunodeficiency virus" AND "aids" OR "acquired immunodeficiency virus syndrome" AND "cross sectional" "india" KNOWLEDGE OR "ATTITUDE" OR "PRACTICE" OR "behavior""knowledge" "attitude" "practice" "questionnaire" OR "survey"

Table S2. List of excluded studies and reasons for exclusion

Article	Reason
Knowledge and attitude of Basic Health Workers (BHWs) toward HIV/AIDS	Not in India
South Asian migrant women and HIV/STIs: knowledge, attitudes and practices and the role of sexual power	Not in India
Knowledge and practices related to sexually transmitted infections and HIV among sex workers in an urban area of Gujarat, India	Full-text not accessed
Knowledge and Perception among Medical Students towards People with HIV and AIDS	Full-text not accessed
Knowledge, Attitudes and Practices Towards HIV/AIDS in General Population Covered by Urban Health Training Centre, Hapur	Full-text not accessed
A Study of Knowledge Regarding HIV/AIDS among the High School Teachers in Tumkur Taluk, Karnatka	Full-text not accessed
Comprehensive Knowledge on Dental Management of HIV/AIDS: A Survey on Private Sector Oral Health Care Providers in Andhra Pradesh	Full-text not accessed
Perceptions about training and knowledge of HIV/AIDS ethics among healthcare providers	Full-text not accessed
Knowledge, attitude, and perception of disease among persons living with human immunodeficiency virus/acquired immuno deficiency syndrome: A study from a tertiary care center in North India	Duplicate article
Knowledge of human immunodeficiency virus, attitudes, and willingness to conduct human immunodeficiency virus testing among Indian dentists	Duplicate article
Knowledge and attitude about HIV/AIDS among HIV-positive individuals in Davangere	Duplicate article
Knowledge and awareness of HIV/AIDS among students of a technical institution	Duplicate article
Knowledge and attitudes about HIV/AIDS of students in HP Government Dental College and Hospital, Shimla, India	Duplicate article
Knowledge, attitude, and behavior in managing patients with HIV/AIDS among a group of Indian dental students	Duplicate article

Figure S1. Meta-regression

a. Knowledge about HIV/AIDS

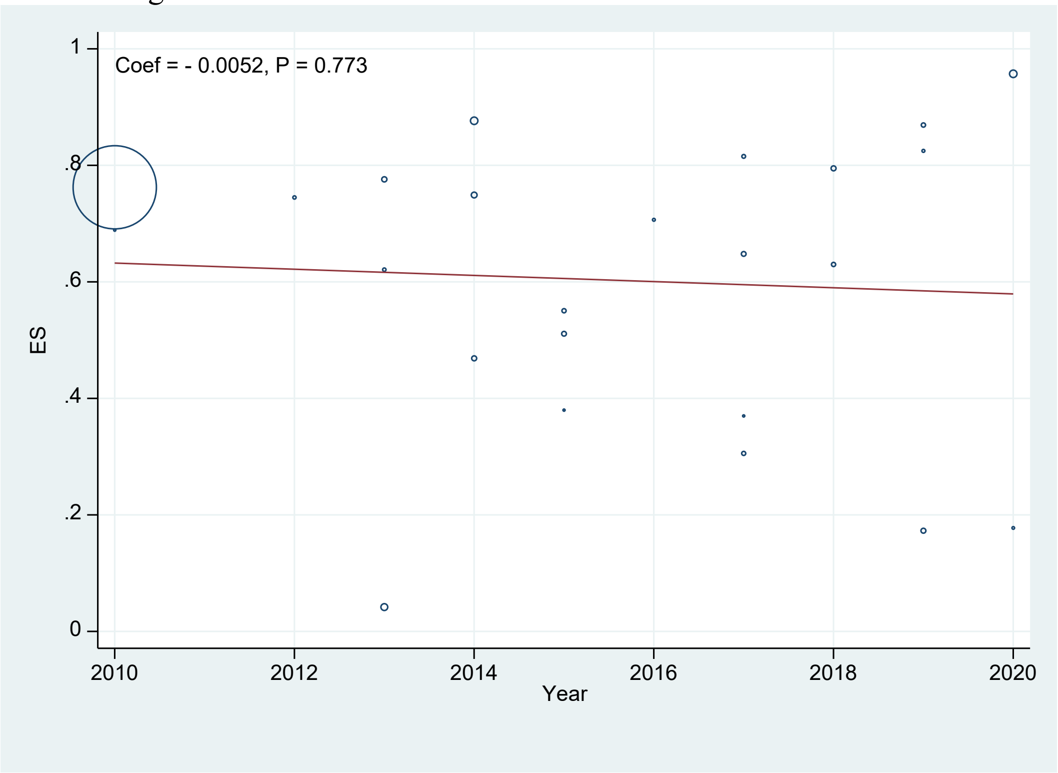

b. Attitude towards HIV/AIDS

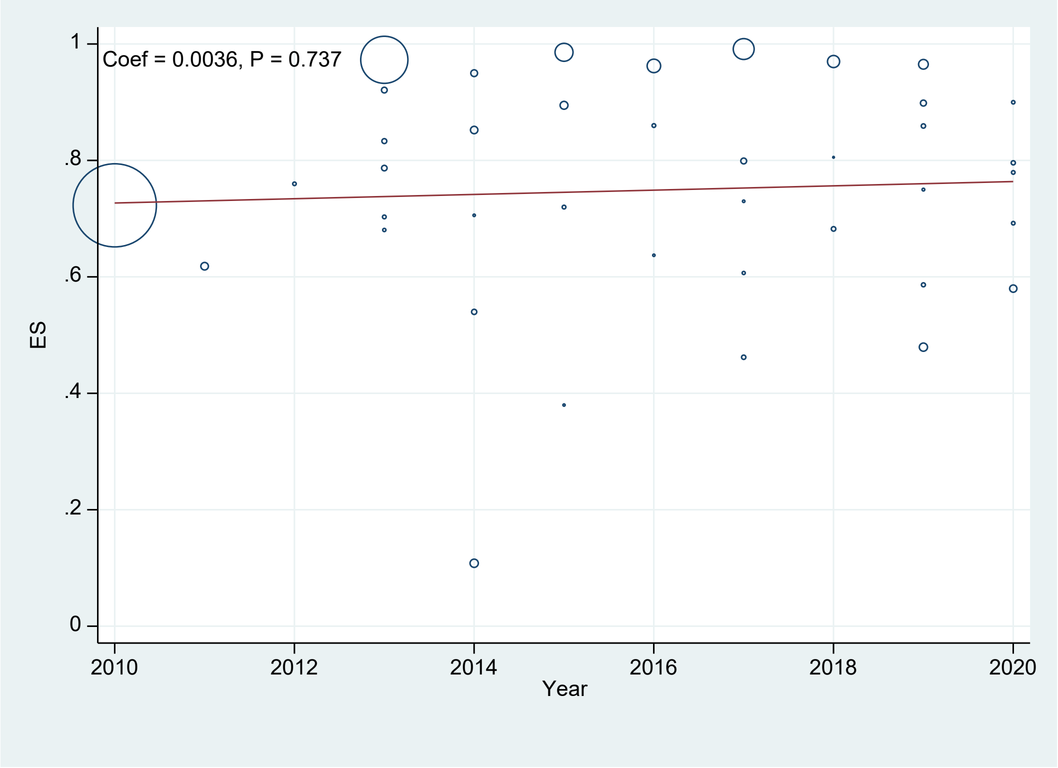

Figure S2. Sensitivity analysis

a. Knowledge about HIV/AIDS

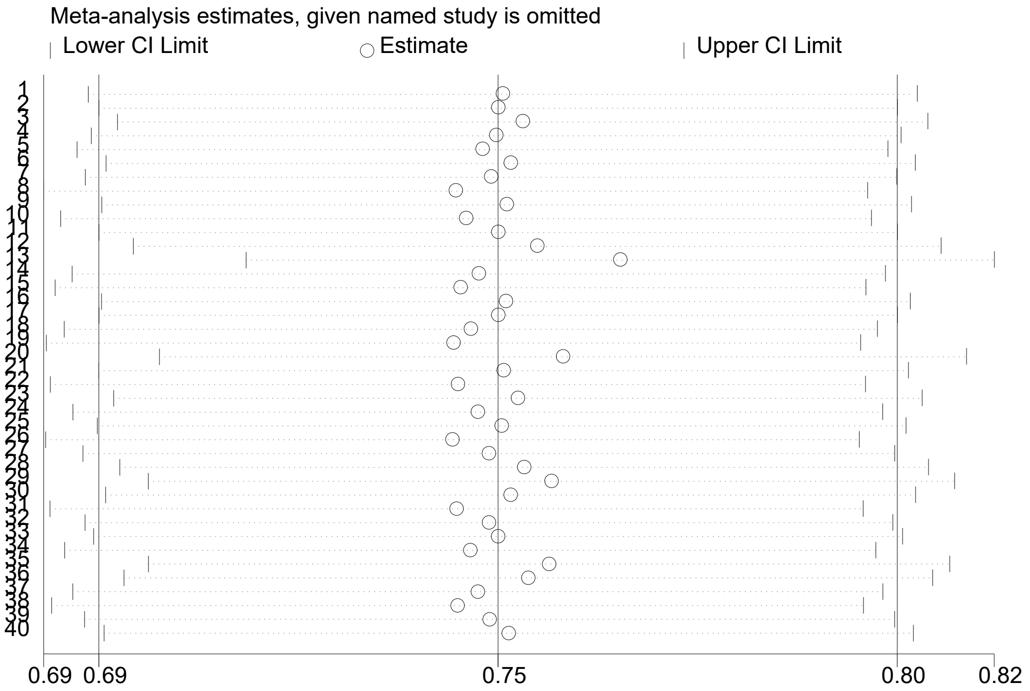

b. Attitude towards HIV/AIDS

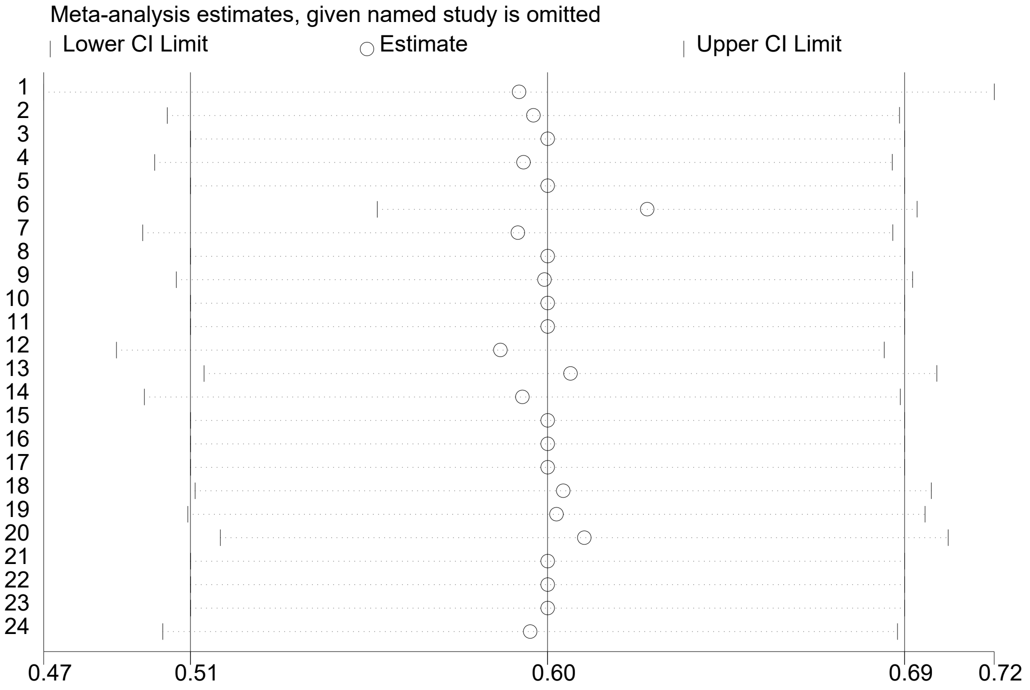

Figure S3. Trim and Fill Publication bias

a. knowledge about HIV/AIDS

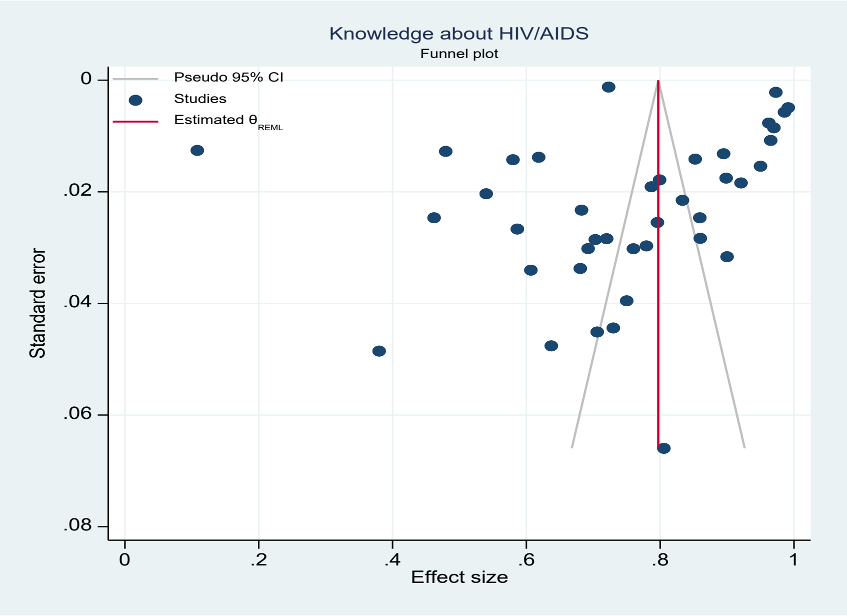

b. Attitude towards HIV/AIDS

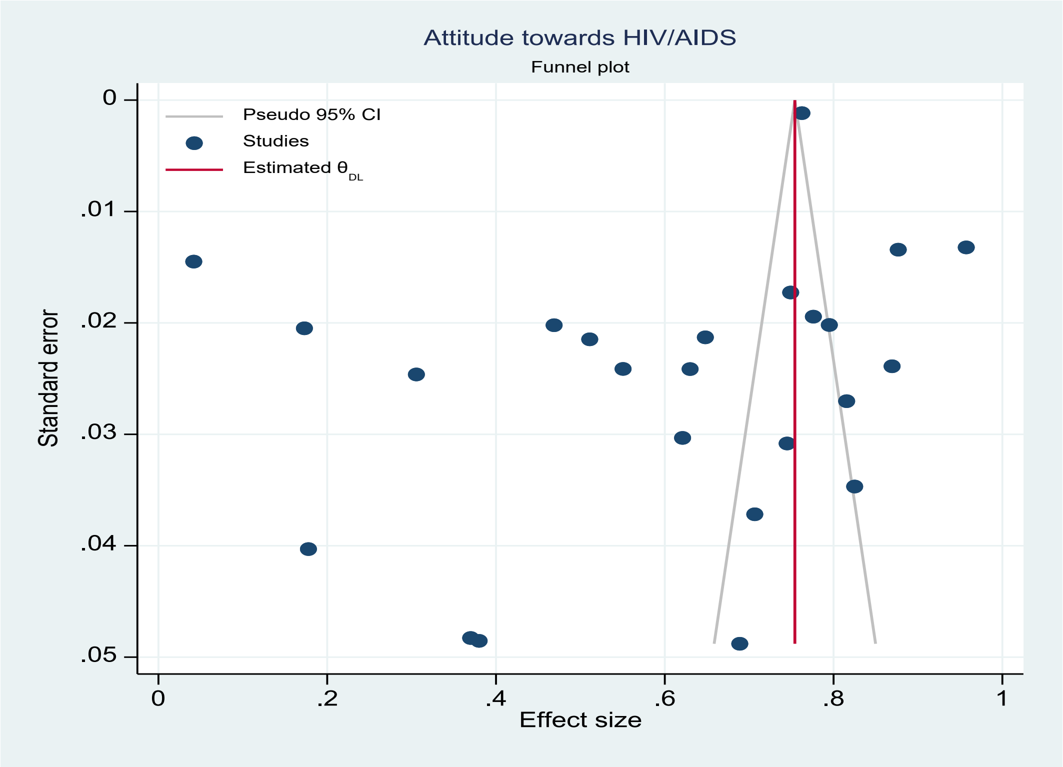

Supplement: Supplementary file 1 — Online Supplementary file 1 contains Tables S1-S2 and Figures S1-S3. [file hpp-11-148-s001.pdf]
